# Effects of Cognitive Load on Pure-Tone Audiometry Thresholds in Younger and Older Adults

**DOI:** 10.1097/AUD.0000000000000812

**Published:** 2019-11-05

**Authors:** Antje Heinrich, Melanie A. Ferguson, Sven L. Mattys

**Affiliations:** 1 Manchester Centre for Audiology and Deafness, University of Manchester, Manchester, United Kingdom; 2 National Institute for Health Research Nottingham Biomedical Research Centre, Nottingham University Hospital’s National Health Service Trust, Nottingham, United Kingdom; 3 National Acoustic Laboratories, Sydney, Australia; 4 Department of Psychology, University of York, York, United Kingdom.

**Keywords:** Aging, Cognitive load, Divided attention, Pure-tone audiometry

## Abstract

Cognitive load (CL) impairs listeners’ ability to comprehend sentences, recognize words, and identify speech sounds. Recent findings suggest that this effect originates in a disruption of low-level perception of acoustic details. Here, we attempted to quantify such a disruption by measuring the effect of CL (a two-back task) on pure-tone audiometry (PTA) thresholds. We also asked whether the effect of CL on PTA was greater in older adults, on account of their reduced ability to divide cognitive resources between simultaneous tasks. To specify the mechanisms and representations underlying the interface between auditory and cognitive processes, we contrasted CL requiring visual encoding with CL requiring auditory encoding. Finally, the link between the cost of performing PTA under CL, working memory, and speech-in-noise (SiN) perception was investigated and compared between younger and older participants.

**Design:**

Younger and older adults (44 in each group) did a PTA test at 0.5, 1, 2, and 4 kHz pure tones under CL and no CL. CL consisted of a visual two-back task running throughout the PTA test. The two-back task involved either visual encoding of the stimuli (meaningless images) or subvocal auditory encoding (a rhyme task on written nonwords). Participants also underwent a battery of SiN tests and a working memory test (letter number sequencing).

**Results:**

Younger adults showed elevated PTA thresholds under CL, but only when CL involved subvocal auditory encoding. CL had no effect when it involved purely visual encoding. In contrast, older adults showed elevated thresholds under both types of CL. When present, the PTA CL cost was broadly comparable in younger and older adults (approximately 2 dB HL). The magnitude of PTA CL cost did not correlate significantly with SiN perception or working memory in either age group. In contrast, PTA alone showed strong links to both SiN and letter number sequencing in older adults.

**Conclusions:**

The results show that CL can exert its effect at the level of hearing sensitivity. However, in younger adults, this effect is only found when CL involves auditory mental representations. When CL involves visual representations, it has virtually no impact on hearing thresholds. In older adults, interference is found in both conditions. The results suggest that hearing progresses from engaging primarily modality-specific cognition in early adulthood to engaging cognition in a more undifferentiated way in older age. Moreover, hearing thresholds measured under CL did not predict SiN perception more accurately than standard PTA thresholds.

## Abstract 2

Supplemental Digital Content is available in the text.

## INTRODUCTION

Listening involves synergies and trade-offs between hearing and cognition. For example, deficiency at the periphery of the auditory system may lead to increased demands on attention and working memory, as well as elevated listening effort (Downs 1982; Rönnberg et al. 2013; see McGarrigle et al. 2014 and Pichora-Fuller et al. 2016, for reviews). In such conditions, cognitive resources might be recruited to bridge gaps in the impoverished auditory input. Consequently, improving peripheral hearing, through hearing aid technology for instance, could allow cognitive spare capacity to be redeployed to other tasks, and hence, reduce cognitive load (CL), listening effort, and fatigue (Sarampalis et al. 2009; Hornsby 2013; Ng et al. 2014; Qian et al. 2016).

The role of cognition in hearing is also supported by the finding that high cognitive functioning, as measured by psychometric tests, is associated with good auditory performance. For instance, individuals with better cognitive abilities, especially among older or hearing-impaired individuals, show better speech-in-noise (SiN) performance (Avivi-Reich et al. 2014) and greater benefit from hearing aids than individuals with poorer cognitive abilities (Lunner & Sundewall-Thorén 2007; Souza & Sirow 2014). The contribution of cognitive resources to the listening experience is also demonstrated in dual-task paradigms. This line of research involves listening to speech while simultaneously performing a cognitively demanding task (e.g., a visual search). CL has been shown to affect various aspects of the listening experience, such as convergence in conversation (Abel & Babel 2017), word segmentation (Mattys et al. 2009; Fernandes et al. 2010), lexical activation (Mattys & Wiget 2011; Zhang & Samuel 2015), speech/noise segregation (Francis 2010; Zekveld et al. 2011), and duration, intensity, and F0 discrimination (Chiu et al. 2019). Of particular interest for the present study is the finding that the disruptive effect of CL on speech processing can be traced as early as in the initial stages of acoustic-to-phonetic encoding, where acoustic features are combined into phonetic features, and phonetic features into phonemes (Mattys et al. 2014; Mitterer & Mattys 2016; Gennari et al. 2018). Under some circumstances, CL has been shown to even lead to what is referred to as inattentional deafness, a form of brief distraction-induced hearing loss (Macdonald & Lavie 2011; Molloy et al. 2015).

The idea of load-related hearing loss is important because it suggests that CL may compromise the basic ability to detect sounds played at low intensity level (Sörqvist et al. 2012; Molloy et al. 2015), which, in turn, can have consequences for everyday communication. In clinical settings, hearing thresholds are measured by pure-tone audiometry (PTA). It is interesting that PTA is thought to measure primarily sensory-neural ability and is considered to be broadly tolerant to extrinsic nonauditory distraction. Therefore, any evidence for an increase of PTA thresholds under CL would suggest that low-level auditory sensation is affected by cognitive demands, as implied by Macdonald and Lavie (2011) and Molloy et al. (2015), and consequently, that PTA is not as cognition-free, a measure of hearing as generally assumed.

There is preliminary evidence that mental workload affects hearing sensitivity. Baldwin and Galinsky (1999) reported elevated PTA thresholds for .5, 1, 2,3, 4, 6, and 8 kHz pure-tones in young, normal-hearing participants who took a PTA test while playing a visually and attentionally demanding computer game. The cost of playing the game ranged from 0.6 to 3.8 dB HL, depending on the ear and the frequency. The implications of that finding are limited, however. First, the methodological details provided in the published report are insufficient to establish how the thresholds were calculated and the extent to which the decrease in sensitivity under CL generalized across individuals. Second, while computer games have reasonable external validity as CL, they do not provide a sufficient basis for determining the locus and mechanisms of interference between CL and hearing sensitivity.

The main aim of the present study was to quantify the detrimental effect of CL on PTA and to understand how the effect of CL on PTA is affected by age. This is a necessary consideration for cognitive approaches to listening because the relation between hearing and cognition is believed to be modulated by age-related sensory and cognitive decline. For example, in a meta-analysis of 41 datasets, Füllgrabe and Rosen (2016) showed that the association between good working-memory capacity and good SiN perception typically found in older or hearing-impaired individuals (Akeroyd 2008) was negligible in young, normal-hearing individuals.

Therefore, following up on Füllgrabe and Rosen’s (2016) analysis, a question for this study was whether older listeners are more likely to experience disruptive effects of CL on PTA than younger listeners. On the one hand, it is possible that the contribution of cognition to listening found in older adults only applies to listening tasks involving complex linguistic processes taking place over durations longer than brief tones or to tasks involving discrimination of complex spectro-temporal details. If so, the effect of CL on PTA should be small, and equally so in younger and older listeners. On the other hand, insofar as PTA requires focused attention and insofar as attention suffers from age-related cognitive decline (Kramer & Madden 2008; Drag & Bieliauskas 2010), older adults should be at a disadvantage when performing PTA under CL. Common to various theories accounting for this expected age difference is the claim that there is a limit on the attentional resources one possesses (Kahneman 1973) and that this limit decreases with age (Craik & Byrd 1982). Therefore, the cumulative demands required by the PTA task and the CL task could be particularly taxing for older adults.

A related area in which the interaction between hearing and cognition has been debated concerns the extent to which PTA can predict SiN perception. PTA has met limited success as a measure of everyday listening abilities (Henshaw et al. 2015; Heinrich et al. 2016). Indeed, clinical practice indicates that individuals who report difficulties understanding SiN do not always show abnormal PTA profiles (Hind et al. 2011). In comparison, listening tests that include a cognitive component have proved to be better predictors (Lunner & Sundewall-Thorén 2007; Heinrich et al. 2015, 2016), probably because understanding sentences in noise engages attentional control and temporary storage of degraded speech in working memory (Dryden et al. 2017). Therefore, a second aim for this study is to understand whether a PTA test that has a cognitive component built into it (e.g., the cost of performing PTA under CL) is a better predictor of SiN perception than PTA alone.

Finally, in an attempt to identify the type of resources that could mediate detrimental effects of CL on PTA, we contrasted CL that requires purely visual encoding with CL that requires auditory encoding. In the former, meaningless, un-nameable images were shown briefly, one after the other, throughout the PTA test. Participants pressed a button every time they saw the repetition of an image that was presented two images before, regardless of its orientation. In the latter, the same two-back procedure was followed, except that the images were replaced with written nonwords and participants pressed a button every time a nonword rhymed with the nonword seen two nonwords before, regardless of its spelling. Although both tasks require maintaining and updating visual information in working memory (e.g., in Baddeley’s [2000] episodic buffer and central executive), the two-back rhyme task also requires rehearsal of the materials in a subvocal auditory format (e.g., phonological loop). If (1) PTA requires cognitive resources, and (2) those resources are shared amodally with those required by the two-back tasks, the encoding format of CL should be irrelevant to the effect of CL on PTA. In contrast, if the resources needed for PTA engage predominantly auditory representations, the detrimental effect of the two-back task should be larger in the rhyme than the image condition. Establishing whether PTA and CL rely on domain-general or domain-specific resources has implications not only for our understanding of cognitive listening and audiological practice, but also for the debate between proponents of a single amodal attentional regulatory system (Duncan & Owen 2000) and proponents of attentional systems devolved to specific modalities (Petersen & Posner 2012).

In this study, we compared PTA with and without CL in younger and older participants. To offer a realistic and representative picture of our older group, we did not attempt to restrict our sample to unusually good hearers. Instead, our sample included participants with a variety of hearing levels, from normal to uncorrected moderate hearing loss. A drawback of this naturalistic approach is that age and hearing level are likely to be partly confounded (Cruickshanks et al. 1998). However, insofar as variability in hearing sensitivity is an intrinsic feature of aging, it should be included in any description of age-related changes in listening mechanisms and strategies. Whether the CL pattern uncovered in this study are specific to aging or could potentially be replicated in younger listeners with hearing profiles similar to those of older people will be considered in the Discussion.

In summary, the present study aimed to address the following questions:

1. Does CL lead to a measureable cost on PTA and, if it does, is this cost larger in older than younger adults? An age difference could arise either because age-related sensory decline requires older adults to allocate more cognitive resources to basic listening or because age-related cognitive decline affects resource allocation in tasks requiring divided attention and working memory. Addressing this question required an analysis of how the size of the PTA CL cost trades-off with performance on the CL task itself. Of interest is whether the size of PTA CL cost is more driven by a trade-off between hearing and cognition or by individual differences in available resources. In the former, we would expect listeners who did well on the CL task to have a larger PTA CL cost. In the latter, we would expect listeners who did well on the CL task to have a smaller PTA CL cost.
2. Is the effect of CL on PTA modulated by the type of CL? Insofar as the memory and attentional resources required during hearing and listening are amodal and domain-general, all types of CL should have the same effect on PTA, whether they involve auditory or visual representations. However, if the resources needed for PTA are encapsulated within the auditory modality, CL involving auditory representations should have a greater effect on PTA.
3. Does PTA CL cost predict SiN perception better than does PTA alone? Given that PTA alone is thought to be suboptimal at predicting SiN and that SiN involves cognitive processes, we expect that PTA CL cost should more accurately represent the sensory-cognitive challenges experienced in noisy speech conditions than PTA alone.

## MATERIALS AND METHODS

### Participants

Forty-four young adults (average age: 21 years, range 18 to 31; 29 female) and 44 older adults (average age: 68 years, range 60 to 84; 25 female) were recruited via local newspaper advertisements and through the University of York participant pool. They received course credit or a small honorarium for their participation. Older participants also received reimbursement for their travel expenses. All were native speakers of British English and reported normal or corrected vision. Hearing levels, which were assessed as part of the main experiment, are reported in the Results section and in the Table in Supplemental Digital Content, http://links.lww.com/EANDH/A584. Thresholds averaged across 0.5, 1, 2, and 4 kHz in the right ear ranged from −8.50 to 13.75 dB HL for the younger group (average 0.64 dB HL) and from −5.00 to 53.50 dB HL for the older group (average 15.16 dB HL). These figures are broadly consistent with, though perhaps slightly better than average population data for the respective two groups (Lee et al. 2005; Wilson & McArdle 2013). None of the participants wore hearing aids.

Participants within each age group were assigned to the image CL condition (N = 22) or the rhyme CL condition (N = 22) alternatively as they came in to the laboratory for testing. Approval for performing the testing described later was granted by the University of York Departmental Ethics Committee, application #215, on March 13, 2015, and followed the guidelines of the 48th World Medical Assembly (1997).

### Stimuli

#### Image and Rhyme Stimuli for the CL Tasks •

The stimuli used in the image CL condition were black and white line drawings taken from Kroll and Potter (1984). These depicted meaningless and non-nameable objects. The stimuli in the rhyme CL condition were written monosyllabic nonwords conforming to the phonotactic constraints of British English. They were selected in part from the ARC Nonword Database (Rastle et al. 2002). All nonwords consisted of an onset, a nucleus, and a coda. Onsets and codas could be made of a single consonant or multiple consonants. Nuclei could be any English vowel or English diphthong.

#### Auditory Stimuli for the SiN Tasks •

The stimuli for the SiN tasks were meaningful, semantically neutral, and phonemically balanced sentences drawn from the IEEE corpus (Rothauser et al. 1969), e.g., Glue the paper to the dark blue background. Several sentences were modified to conform to contemporary British English. The sentences were recorded by a native British English male speaker. Average sentence duration was 2032 msec (range: 1269 to 2868 msec). The background noise consisted of 2- and 12-talker babble noise and their speech-modulated noise (SMN) equivalent, henceforth labeled 2Babble, 12Babble, 2SMN, and 12SMN. These four types of noise were meant to provide a range of difficulty levels. All babble speakers were native English speakers and were recorded individually while reading a piece of prose of their choice. Although the four types of noise differ in terms of energetic and informational masking (Koelewijn et al. 2012; Rosen et al. 2013; Zekveld et al. 2013), performance was averaged across all four conditions in all analyses for simplicity. Their average is subsequently denoted as “SiN performance.”

Sampling rate was 22.05 kHz. All single-voice recordings of the babble had equal long-term root mean square amplitude values to ensure that no voice stood out. The SMN conditions were created in Matlab (R2014b) by averaging the babble signal in chunks of 23 msec. This preserved the long-term average spectrum of the signal as well as the overall signal envelope, but it made the sound unintelligible. All sound signals were low and high pass filtered between 50 and 10,000 Hz.

### Design and Procedure

**Fig. 1.**
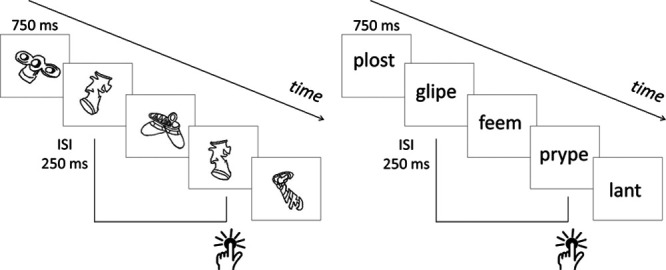
Sequence of tasks during the experiment. CL indicates cognitive load (image or rhyme); LNS, letter number sequencing; PTA, pure-tone audiometry; SiN, speech-in-noise.

Participants followed the sequence of tasks shown in Figure 1. Within each age group, participants were assigned to either an image or a rhyme CL condition. We chose a between-subjects design to keep the number of PTA tracks administered to each participant to a manageable number and to minimize practice effects across tracks. All other variables were administered within participants. In total, the experiment lasted between 60 and 90 minutes. Short breaks were allowed between tests.

**Fig. 2.**
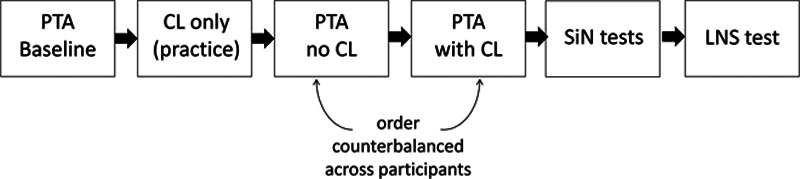
Illustration of the two-back task used as CL during the pure-tone audiometry test. Image CL (left) and rhyme CL (right). An example of a two-back repetition of an image or a rhyme is shown by the button-press symbol. CL indicates cognitive load.

#### PTA Baseline •

Participants started with a baseline PTA test (PTA-Basl), in which PTA was measured without CL. PTA-Basl acted as practice and was meant to reduce test–retest improvement in the subsequent blocks. It was also used to determine the test–retest reliability of our main outcome measure, PTA. We assessed test–retest reliability using the intraclass correlation coefficient (ICC) following Weir’s (2005) recommendations.

PTA-Basl was administered manually, broadly following the British Society of Audiology (2011) recommended procedures, over TDH49 headphones and an AD226 Interacoustics diagnostic audiometer. To minimize testing time, PTA was performed on the right ear only. The right ear was chosen because it was ipsilateral with the hand used to indicate tone detection. Pure-tones were presented in the following order: 1, 2, 4, 0.5 kHz. To indicate tone detection, participants pressed a response switch held in their right hand. The PTA test followed a one-up-one-down adaptive staircase procedure, starting at 30 dB HL and decreasing in 10-dB steps until the first reversal. The step size was then 5 dB until the second reversal. Afterward, the downward step size was 6 dB and the upward step size was 2 dB. The test stopped after a given level was used as an up-down reversal twice, consecutively or not, within the 6-2 dB part of the track. That level was used as the PTA threshold.

#### Practice CL Task •

After the PTA-Basl test, participants practiced the CL task without PTA. This block was meant to familiarize participants with the CL task before they had to perform it simultaneously with the PTA test. Each visual stimulus (image or nonword, depending on the participant’s group assignment) was displayed for 700 msec, with a 250-msec interstimulus interval. On two-back image repetition trials, the same image was presented, rotated 30° to the left or to the right (see Figure 2 for an illustration). In the rhyme condition, two-back rhyming nonwords shared their rhyme. To encourage phonological encoding, the nonwords of some rhyming pairs differed in their spelling (e.g., glipe, prype). Foil pairs were also included. These consisted of nonrhyming pairs that shared their nucleus (e.g., swease, feame) or their onset and nucleus (e.g., swease, sweake). Participants were instructed to press a key on a computer keyboard with the left hand every time they saw an image that was the same as the image that appeared two images before, or every time they saw a nonword that rhymed with the nonword presented two nonwords before. During the rhyming task, participants were not allowed to rehearse the syllables out loud. Participants were also told that repeated images would not be in the same orientation and that rhyming nonwords would not necessarily be spelled the same way. The practice CL block contained 20 instances of two-back image repetitions or rhymes. Image repetitions or rhymes occurred every six stimuli on average.

#### PTA With and Without CL •

After practicing the CL task, participants did the two key blocks: PTA without CL (PTA-NoCL) and PTA with image or rhyme CL (PTA-CL). PTA-NoCL was procedurally identical to PTA-Basl. In the PTA-CL condition, the CL task followed the procedure described for the practice CL block; the PTA and CL tasks started and ended at the same time. The total number of images or nonwords displayed in the PTA-CL conditions depended on the duration of the concurrent PTA test. The order of PTA-NoCL and PTA-CL was counterbalanced across participants.

#### SiN Tasks •

Next, participants did 4 SiN tests in the following order: 2Babble, 2SMN, 12Babble, 12SMN. This order was chosen arbitrarily and it was the same for all participants. For each of the four conditions, maskers were sampled from a 2-min uninterrupted recording of that masker type. The sampled portion of the 2-min recording was chosen randomly for each target sentence. The masker started 2 sec before the target and ended 2 sec after it. Fade-in and fade-out were 10 msec long.

In each SiN condition, participants heard 20 different target sentences. Each sentence contained five key words (all content words), which were used as a basis for establishing the speech reception threshold (SRT). The 4 sets of 20 sentences were rotated across SiN conditions in quadruplets of participants. Target sentences and maskers were played binaurally. The target sentences were played at 65 dB SPL. Following a one-up-one-down adaptive staircase procedure, the first masker within a track was played at +10 dB SNR, decreasing in 10-dB steps until the first reversal. The step size until the second reversal was 5 dB. The step size was 2.5 dB subsequently. On each trial, participants were asked to repeat the target sentence as accurately as possible. SNR decreased if participants correctly reported three or more key words out of five. SNR increased if they correctly reported two or fewer keywords. SRT therefore corresponded to 50% accuracy. The track stopped after either 6 reversals or 20 trials, whichever came first. SRT was measured as the average of all the SNR values within the 2.5-dB step size part of the track, whether or not those values corresponded to reversal points. SiN performance was measured as the average SNR across 2Babble, 2SMN, 12Babble, and 12SMN.

#### Letter Number Sequencing Task •

Finally, participants did the letter number sequencing (LNS) task (Wechsler 1997), which is a measure of verbal working memory that includes a strong executive component. On each trial, participants heard a sequence of prerecorded numbers and letters (e.g., 4-S-6-A), which they were instructed to recall, repeating the numbers in ascending order first, followed by the letters in alphabetical order (e.g., 4-6-A-S). Sequences began with two items (e.g., L-2, repeated as 2-L) and increased to a maximum of eight items. For each sequence length, three different trials were presented, of which at least one had to be correctly recalled to advance to the next, longer sequence. The task was terminated when none of the three trials within a sequence length was recalled correctly. The sum of correctly recalled sequences was used as the outcome measure.

### Modeling

All statistical analyses were run in R version 3.3.2 (2016-10-31) (R Core Team 2013), using RStudio 0.99.489 and the following packages: lme4 (Bates et al. 2015), lsmeans (Lenth 2016), lmerTest (Kuznetsova et al. 2016), lmtest (Zeileis & Hothorn 2002), and multcomp (Hothorn et al. 2008).

To assess the effect of CL on PTA, we set up a general linear mixed model (GLMM) with PTA threshold as the outcome measure. Fixed-effect predictors were two between-subject factors, namely, age group (younger, older) and CL type (image, rhyme), and two within-subject factors, namely, CL (NoCL, CL) and frequency (0.5, 1, 2, 4 kHz). All fixed effects except frequency were coded as categorical variables. Participants, load-by-participants, and frequency-by-participants were random terms, which mirrored the random effect structure of a mixed-measures analysis of variance.

To compare the predictive effects of PTA under NoCL and under CL on SiN performance, we ran a GLMM with the following parameters. The outcome variable was the SNR achieved by the listener. PTA measured under no load (NoCL) and PTA CL cost (the difference between PTA under NoCL and under CL), both averaged across the four frequencies, were within-subject continuous predictors. Age group (younger, older) was a between-subject categorical predictor. The most efficient random effect structure included only participant effects.

In both analyses, the final model was determined using a backward stepwise procedure, as suggested by Pinheiro and Bates (2000). Following this procedure, the original full model, which included all main effects and interactions, was pruned so that the final model included higher-level interaction terms only if they improved model fit. Model fit was estimated by the Akaike Information Criterion (AIC), an index of the relative fit of a mixed model. The AIC values of the model with and without a particular term were compared. If the simpler model led to a reduction in AIC, it was adopted. If the simpler model led to an increase in AIC, the AIC values of the two models were compared by means of a χ^2^ test. Only when the more complex model led to a significantly better fit was the higher-level interaction kept in the model. The principle of marginality was observed such that if a higher-level interaction was kept in the model, the nested lower-level (subordinate) interactions were also retained without testing. For example, if A × B × C was kept in the model, the model also included A × B, A × C, and B × C. Main effects were always kept in the model. During the stepwise procedure, we used ML estimation. Once the final model was established, the fixed effects were calculated using REML estimation and Type 3 SS with Satterthwaite approximation for degrees of freedom. This modeling approach has been previously used by Knight and Heinrich (2017, 2018).

Posthoc testing for categorical variables was conducted using lsmeans with Bonferroni correction. Posthoc testing for interactions involving continuous variables (NoCL, PTA CL cost) was conducted by rerunning the model with the dataset divided along the critical categorical variable to determine the extent to which the significant interaction including the continuous variable generalized across all levels of the categorical variable.

In some analyses, performance on the two-back CL task was entered as a continuous predictor. Two-back performance was calculated as the ability to discriminate between a repeated image (or rhyme) and a nonrepeated one using *d*’ from signal detection theory (Green & Swets 1966). *d*’ values were centered across the two age groups by subtracting the combined mean of the two groups from each score. This was done to preserve the group difference while setting the intercept to a common, normalized mean, in accordance with Schneider et al.’s (2015) recommendation regarding analysis of covariance (ANCOVAs).

## RESULTS

### Effects of CL on PTA

**Fig. 3.**
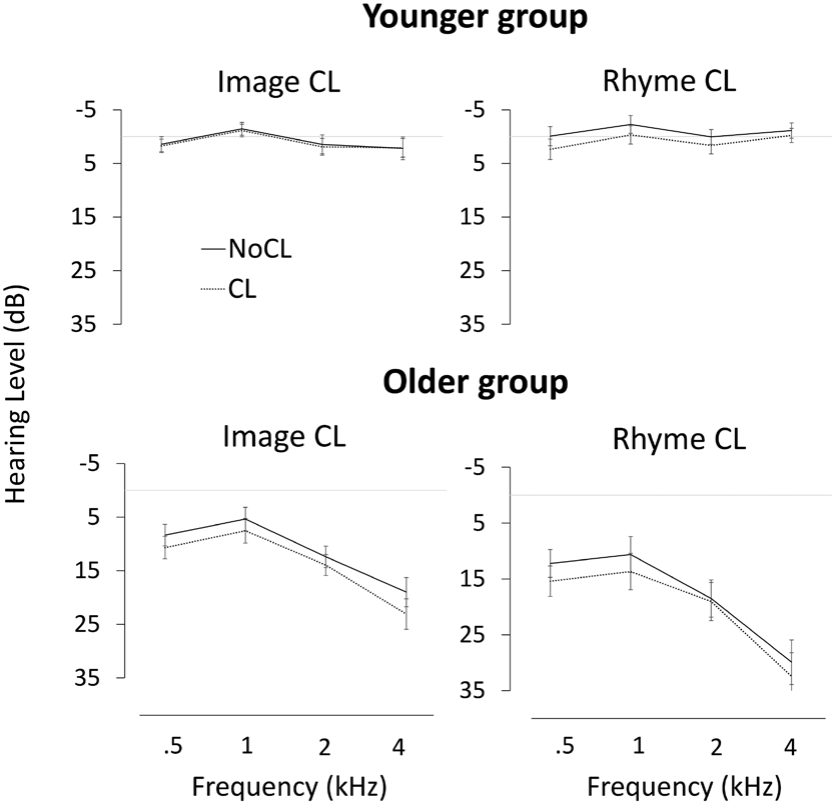
Hearing threshold levels (dB HL) and standard error of the mean for younger and older participants as a function of frequency (0.5, 1, 2, 4 kHz), CL (NoCL vs. CL), and CL type (image vs. rhyme). CL indicates cognitive load.

Individual and group data for all variables are reported in the Table in Supplemental Digital Content, http://links.lww.com/EANDH/A584. Figure 3 shows average PTA by CL (NoCL, CL), frequency (0.5, 1, 2, 4 kHz), CL type (image, rhyme), and age group (younger, older). First, we assessed the equivalence in basic hearing levels (PTA-Basl) between the participants assigned to the image CL condition and the rhyme CL condition. The two younger groups were comparable (image: 1.2 dB; rhyme: 0.1 dB), *F*(1, 42) < 1. The older participants in the image group had marginally better hearing than those in the rhyme group (image: 11.9 dB; rhyme: 18.5 dB), *F*(1, 42) = 3.57, *p* = 0.07.

The main question was whether CL worsened PTA thresholds and, if it did, whether this effect was modulated by age and the type of CL. GLMM analyses showed significant main effects of CL, *F*(1, 86) = 33.35, *p* < 0.001, frequency, *F*(3, 258) = 28.28, *p* < 0.001, and age group, *F*(1, 84) = 65.56, *p* < 0.001. Significant interactions were found between CL × Age Group, *F*(1, 86) = 5.96, *p* = 0.02, CL Type × Age Group, *F*(1, 84) = 3.91, *p* = 0.05, Frequency × Age Group, *F*(3, 258) = 21.99, *p* < 0.001, and CL × Frequency × Age Group, *F*(3, 258) = 2.98, *p* = 0.03. Significant effects (*p* < 0.05) and results of Bonferroni-adjusted posthoc tests based on the final model are shown in Table 1.

**TABLE 1.**
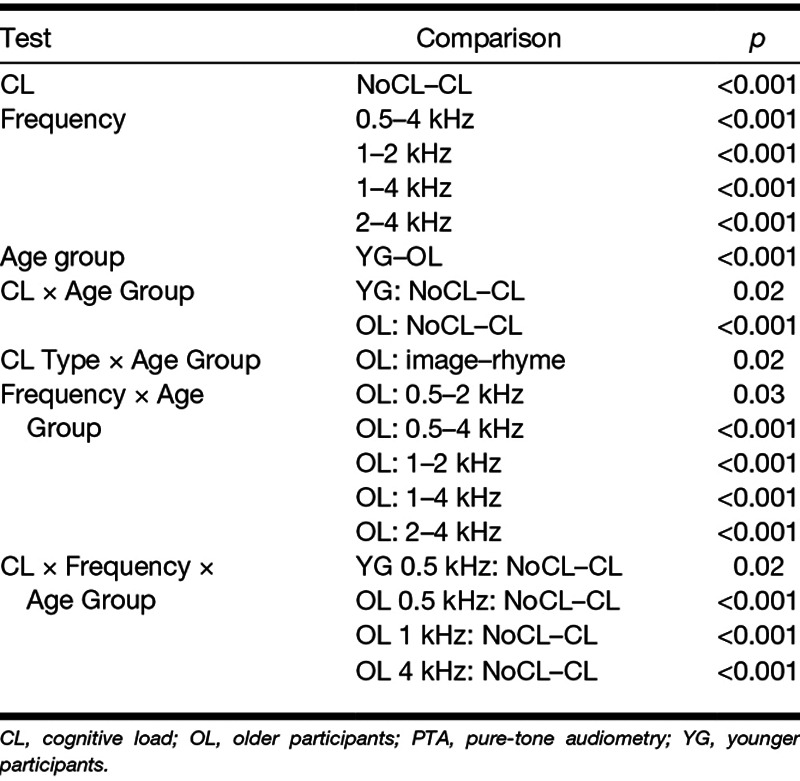
Significant comparisons based on the final model testing the effects of CL, frequency, age group, and CL type on PTA

These tests show the expected age effect on PTA, with younger listeners having better hearing sensitivity than their older counterparts. Younger listeners displayed a relatively homogenous hearing profile across frequencies, whereas older adults displayed the sloping high-frequency hearing loss typically associated with aging. Critically, CL increased hearing thresholds overall and, as suggested by the CL × Age Group interaction, this increase was greater for older than for younger listeners (2.5 versus 1.0 dB, respectively). The CL × Frequency × Age Group interaction indicates that, in the younger group, only the lowest frequency (0.5 kHz) showed a CL effect, whereas, in the older group, all but one frequency (2 kHz) showed a CL effect.

**Fig. 4.**
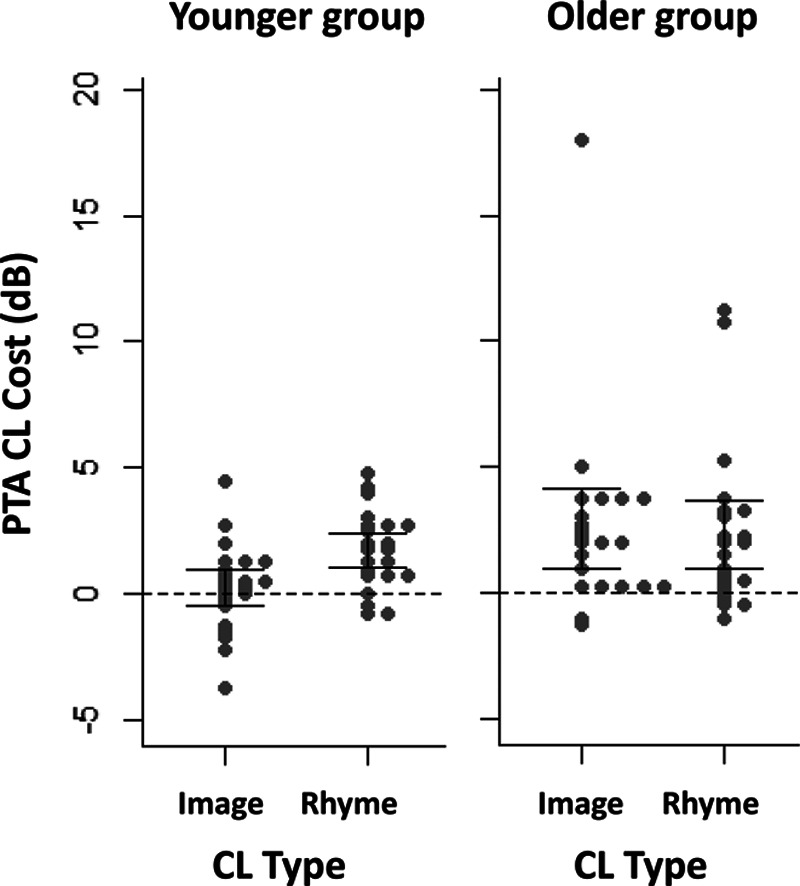
PTA CL cost (dB) as a function of CL type (image, rhyme) and age group (younger, older). Each dot represents an individual participant. For each participant, PTA CL cost was calculated as PTA under CL (dB) minus PTA under no CL (dB). Positive values indicate poorer hearing sensitivity under CL than NoCL. Error bars show 95% confidence interval. CL indicates cognitive load; PTA, pure-tone audiometry.

Although the CL × Age Group × CL Type interaction did not reach significance, *F*(1, 84) = 2.06, *p* = 0.15, a visual inspection of the CL × CL Type pattern in each age group revealed crucial differences that required further investigation. In the younger group, an interaction between CL and CL type, *F*(1, 42) = 8.83, *p* < 0.005, showed that the detrimental effect of CL was observed under rhyme CL (1.7 dB, *p* < 0.001) but not under image CL (0.2 dB, *p* = 0.48). In contrast, that interaction was not significant in the older group, *F*(1, 42) < 1, with a significant CL effect under both image CL (2.5 dB, *p* = 0.002) and rhyme CL (2.3 dB, *p* = 0.004). Figure 4* illustrates this contrast in terms of PTA CL cost (dB), which is calculated as PTA under CL minus PTA under no CL: Younger listeners showed a CL cost in the rhyme condition only, whereas older listeners showed a CL cost in both the image and rhyme conditions (the error bars represent 95% confidence intervals).

We then investigated the relationship between the participants’ PTA and their performance on the two-back CL task. Of interest was whether the deterioration of PTA under CL might be more pronounced in listeners who do well on the two-back task (resource trade-off between PTA and the CL task) or, alternatively, in listeners who do poorly (individual differences in available resources). First, we analyzed performance in the two-back CL task (indexed by *d’*) as a function of CL type (image, rhyme) and age group (younger, older). None of the terms was significant: CL type, *F*(1, 84) = 2.00, *p* = 0.16; age group, *F*(1, 84) < 1, CL Type × Age Group, *F*(1, 84) < 1, suggesting that the two-back performance was comparable across age groups and CL types. We then conducted an ANCOVA on PTA thresholds with all the variables previously modeled (CL, CL type, frequency, and age group) and included participant performance on the two-back task as an additional predictor.

**Fig. 5.**
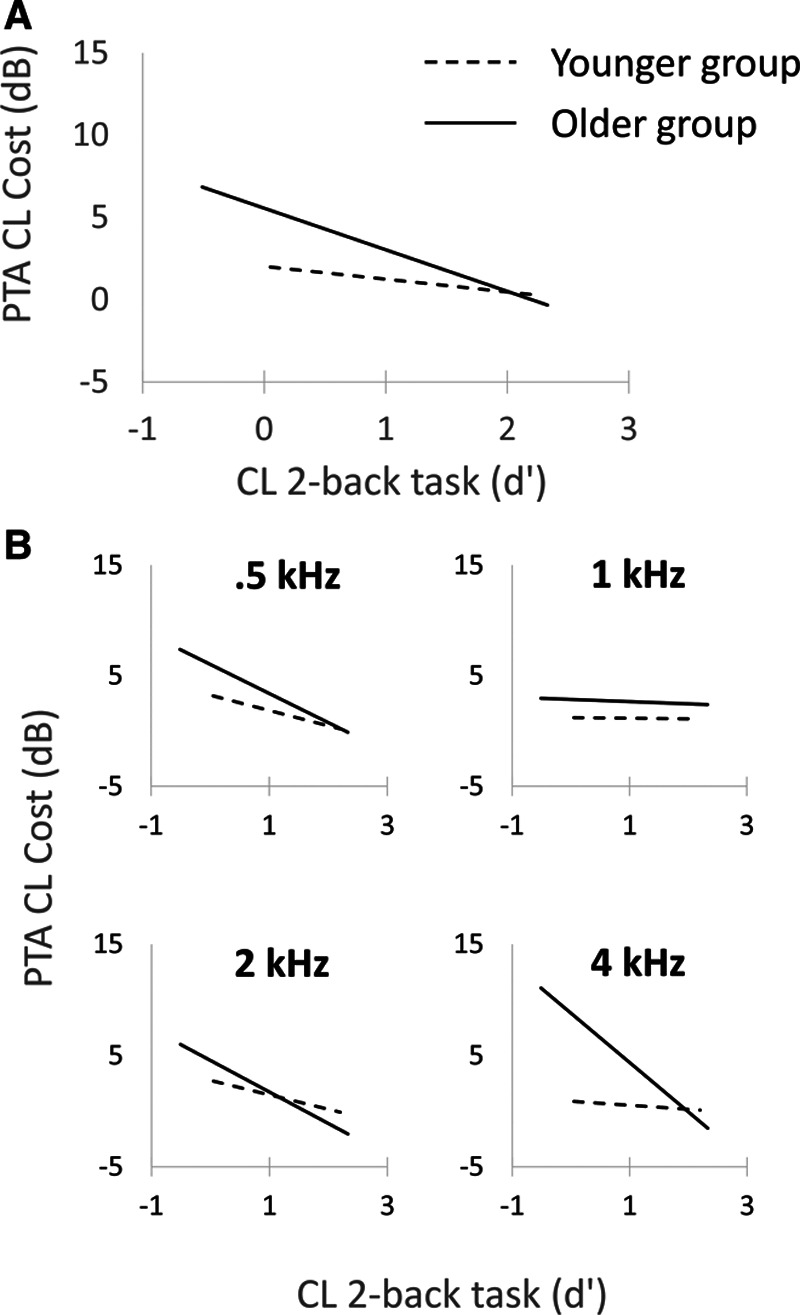
A, Regression lines between PTA CL cost and performance on the CL two-back task for younger and older participants averaged across all frequencies. B, Same data broken down by frequency (0.5, 1, 2, 4 kHz). CL indicates cognitive load; PTA, pure-tone audiometry.

The ANCOVA showed the same main effects and interactions as in the main analysis. In addition, there were two interactions involving two-back performance: CL × two-back Performance, *F*(1, 83) = 9.88, *p* = 0.002, and CL × Frequency × two-back Performance, *F*(3, 249) = 3.69, *p* = 0.01. For consistency with the previous analyses, we explored these interactions separately for the two age groups. ANCOVAs showed that these interactions were only significant in the older group: CL × two-back Performance, *F*(1, 41) = 8.44, *p* = 0.006 (younger group, *F*[1, 41] < 1) and CL × Frequency × two-back Performance, *F*(3, 123) = 3.46, *p* = 0.02 (younger group, *F*[3, 123] < 1). Thus, older participants with poor two-back performance experienced greater PTA cost under CL. This was true at all tested frequencies except 1 kHz, which showed no PTA CL cost (Fig. 5). In sum, for older participants, poor performance on the CL task was broadly associated with a larger PTA CL cost whereas this association was absent in the younger group.

### Link Between PTA CL Cost and SiN Performance

In the following analyses, we asked whether the magnitude of PTA CL cost predicts SiN performance and, if it did, whether it does so better than PTA alone. To decide which variable we should use as a measure of PTA alone, we compared PTA-Basl and PTA-NoCL. As a reminder, these were identical PTA tests, but PTA-Basl was run as practice at the beginning of the experiment to minimize subsequent test–retest effects. PTA-Basl and PTA-NoCL were highly correlated (younger: *r* = 0.94; older: *r* = 0.99). In the younger group, average PTA-Basl was 0.64 dB HL (SD: 5.07) and average PTA-NoCL was 0.02 dB HL (SD: 5.48), *t*(175) = 4.28, *p* < 0.001. In the older group, these values were 15.17 dB HL (SD: 11.80) and 14.55 dB HL (SD: 11.34), *t*(175) = 4.41, *p* < 0.001. The significant difference between PTA-Basl and PTA-NoCL suggests that retest improved performance and, therefore, that PTA-NoCL is a better basis for comparison with PTA-CL.

We used ICC to calculate test–retest reliability coefficients for young and older listeners separately as well as for the combined group. We did this because, while all three data sets showed systematic improvements from the first test to the second test, this improvement was greater for older listeners (2.44 dB) than younger participants (0.99 dB). The ICCs were 0.93 and 0.94, respectively (combined: 0.96). The standard error of the mean were 0.37 and 0.74 dB, respectively (combined: 0.45 dB).

In a first analysis, we modeled SiN performance using PTA-NoCL, working memory LNS, and age group as predictors. Good SiN performance (low SNR) was associated with good hearing (low NoCL PTA), *F*(1, 80) = 7.11, *p* = 0.009, and with younger rather than older listeners, *F*(1, 80) = 6.95, *p* = 0.01. SiN was not predicted by LNS, *F*(1, 80) < 1. However, a marginal 3-way interaction, *F*(1, 80) = 2.79, *p* = 0.09, highlighted contrasted patterns between the younger and older groups. While SiN in the younger group was not predicted by either PTA-NoCL or LNS (*p*s > 0.20), SiN in the older group depended on both PTA-NoCL, *F*(1, 40) = 17.47, *p* < 0.001 (good SiN – good hearing), and an interaction between PTA-NoCL and LNS, *F*(1, 40) = 6.29, *p* = 0.02. The interaction indicated that good SiN performance in the older group was associated with good LNS, and that this was particularly true for older participants with poor hearing compared to good hearing. In sum, this analysis shows that SiN performance can be predicted by hearing thresholds, but that this link is stronger in older than younger listeners. Likewise, the analysis shows that good SiN performance is associated with good working memory scores, but only in older listeners or in listeners with poor hearing thresholds.

In the second analysis, we replaced PTA-NoCL with PTA CL cost. There was no evidence that PTA CL cost predicted SiN performance, *F*(1, 80) < 1, but good SiN performance was associated with good LNS, *F*(1, 80) = 9.00, *p* = 0.004. However, an interaction between LNS and age group, *F*(1, 80) = 4.71, *p* = 0.03, showed that this link was only present in the older group, *F*(1, 40) = 6.96, *p* = 0.01 (younger group, *F*[1, 40] < 1). In sum, contrary to our expectations, PTA CL cost was a comparatively poorer predictor of SiN than PTA-NoCL. Indeed, adding PTA CL cost to a model containing LNS and age group as predictors of SiN did not improve data fit, *χ*^*2*^(1) = 1.11, *p* = 0.29. In contrast, adding PTA-NoCL instead of PTA CL cost did, *χ*^*2*^(1) = 26.01, *p* < 0.001. Furthermore, adding PTA CL cost to a model containing LNS, age group, and PTA-NoCL only marginally improved data fit, *χ*^*2*^(1) = 2.75, *p* = 0.10.

## DISCUSSION

CL has been shown to disrupt various processes involved in speech perception and spoken language comprehension. In this study, we asked whether CL also affects hearing sensitivity and whether age-related sensory and cognitive decline modulates this effect. Younger and older participants performed a PTA test while simultaneously doing a visual two-back task. The stimuli in the two-back task involved either visual encoding (detecting matching images) or subvocal encoding (detecting rhyming nonwords). The PTA results were then analyzed in relation to the participants’ SiN performance and their working memory (LNS task).

Averaged across all groups and conditions, detection thresholds increased by a small but significant amount (1.7 dB) under CL. Before analyzing the modulation of this effect by age and type of CL, we discuss a possible mechanism by which CL might disrupt PTA. This possibility is based on two connected assumptions: (1) CL causes the auditory signal to be under-sampled, and (2) Under-sampling leads to an underestimation of sound intensity. There is empirical evidence for both assumptions. According to the general-timer theory (Macar et al. 1994; Coull et al. 2004), CL forces listeners to rapidly shift attention back and forth between the auditory signal and the CL stimuli, leading to a reduction in the number of samples available to assess the auditory signal. Sample loss would distort auditory temporal judgment, causing listeners to experience “time shrinkage” under CL. Time shrinkage during speech perception was demonstrated by Casini et al. (2009), who reported a consistent underestimation of vowel duration under CL (see also Bosker et al. 2017, for confirmatory evidence).

The link between reduced duration and impaired sound detection, the second assumption, has been documented in psychoacoustic experiments on temporal integration. These have shown that the precision with which listeners judge the loudness of a tone is a function of the duration of the tone, with longer presentations yielding better detection and discrimination (Florentine 1986; Florentine et al. 1988; for a review, see, e.g., Gerken et al. 1990). Poorer performance at shorter durations is thought to result from the smaller number of input samples over which hair-cell neural spikes can be calculated and distinguished from internal noise (see Viemeister & Wakefield’s 1991, multiple-look model). Longer durations allow intensity to be calculated over more neural spikes, and therefore precision of sound detection to be enhanced. Thus, CL-related under-sampling of the auditory signal could plausibly lead to a lack of precision in detecting low-intensity sounds, and hence, cause threshold elevation.

In our experiment, threshold elevation under CL was found in all conditions, except for younger listeners under purely visual CL (two-back image task). To account for this pattern within the under-sampling hypothesis, we propose that sample loss in younger participants only occurs when the representational format of CL competes with that of the PTA task (i.e., both auditory). This would be consistent with a domain-specific view of attentional allocation, which posits that attentional systems are dedicated to specific modalities (Woodruff et al. 1996; Adcock et al. 2000; Petersen & Posner 2012). On that account, processing visual representations during the two-back image task would not interfere with sampling the auditory information during the PTA task because visual and auditory sampling would be handled by separate systems.

The fact that both types of CL representations interfered with PTA in older adults suggests important changes in attentional regulation and sensory representations over the lifespan. As far as auditory sensitivity is concerned, the aging process may involve a leakage of cognitive resources across representational modalities: hearing would rely on modality-specific resources in young adulthood and on more amodal resources later in life. This transformation could be explained either by an age-related change in how representations are organized in short-term memory or by an age-related change in the amount of cognitive resource available to perform simple auditory tasks. In the latter case, the decrease in cognitive resources and attentional control known to happen in older age (Hasher & Zacks 1979; Kramer & Madden 2008; Drag & Bieliauskas 2010) could stimulate competition for resources during divided attention, and this competition may be better handled if allowed to spread across modalities.

The modality-nonspecific CL effect observed in the older group is in line with the finding that response selectivity and brain specialization decrease during the lifespan, a phenomenon known as age-related dedifferentiation (Reinert 1970; Baltes & Lindenberger 1997). For instance, Wong et al. (2009) reported that, compared with young adults, older adults showed more diffused brain activity during SiN perception than younger adults, with evidence that difficult listening involved not only auditory areas but also areas associated with memory and attentional processes. A reason given for the decrease in cognitive and brain specificity in older age is that neurons lose their response selectivity to stimulation, thereby causing additional populations of neurons to be recruited during the activation of a particular representation (Grady 2012). Age-related neural recruitment, compensation, and a decreased ability to inhibit competing responses could be responsible for the older group’s modality-nonspecific response to CL in this study.

The results also indicated individual differences in how participants allocated resources to the two tasks. Older listeners who did poorly on the two-back CL task incurred a larger PTA CL cost during dual-tasking. Younger listeners did not show a significant link between the two tasks. A possible interpretation for this pattern is that, unlike older adults, younger adults were able to perform PTA largely independently from cognition. In contrast, older adults experienced a tighter link between the two—another form of age-related “leakage” of cognitive resources. Interestingly, this link indicated generalization of resource usage (good CL performance – good PTA performance) rather than a trade-off between tasks (good CL performance – poor PTA performance). Thus, older participants with sufficient cognitive resources could successfully allocate those resources to the two tasks, whereas older participants with insufficient cognitive resources struggled with both tasks.

In the analyses linking PTA with SiN performance, we found a strong link between good SiN and good PTA, but no systematic link between SiN and PTA CL cost. Detailed analyses of SiN performance confirmed that the link with PTA was particularly pronounced in older adults. We also found that, among older adults, SiN performance was better for individuals who scored high on the LNS working memory task, and that this relationship was particularly noticeable in individuals with poor PTA thresholds. These results are consistent with Akeroyd’s (2008) and Füllgrabe and Rosen’s (2016) observation that the recruitment of working memory during challenging listening is stronger in older individuals or individuals with hearing loss than in younger, normal-hearing individuals. The fact that the link among SiN, PTA, and LNS was observed in older but not younger adults provides additional evidence to our claim that listening becomes more cognitively dependent over the lifespan.

However, the lack of predictive value of PTA CL cost for SiN, regardless of age, disconfirms our initial hypothesis that a PTA measure that includes a cognitive component is a better predictor of SiN than PTA alone. The possibility that SiN is not an appropriate measure of cognitive listening can probably be rejected based on the finding by Heinrich et al. (2015) that SiN performance, unlike simpler speech perception tasks, is predicted by both PTA and cognitive tests. It is possible, however, that SiN perception did not sufficiently engage the specific aspects of cognition assessed in our study (PTA CL costs and LNS).

An alternative explanation for the lack of relationship between PTA CL cost and SiN is that the type of CL we used was not an appropriate measure of the cognitive processes involved in sentence processing. Cognitive tests known to correlate with SiN involve mostly verbal working memory (Rönnberg et al. 2010; Desjardins & Doherty 2013), particularly those indexing its phonological component (Millman & Mattys 2017). However, the two-back tasks we used as CL met these criteria, especially the two-back rhyme judgment task. Moreover, the relatively low performance on the two-back tasks (1.3 *d*’ on average) suggests that CL was sufficiently difficult to impact on the concurrent task. Thus, it is unlikely that the CL we used was inappropriate for evaluating the cognitive component of sentence perception.

Another possibility is that CL-related PTA threshold elevation was not sensitive enough to capture individual differences in SiN processing. This option should be given serious consideration given the claim made by some (van Rooij & Plomp 1990, 1992) that the contribution of cognition to speech perception in older participants, although real, is relatively small. Likewise, PTA tests, or modifications thereof, might simply not be the right kind of measures for assessing everyday listening, a possibility that has been voiced by hearing researchers (Plomp 1978; Killion & Niquette 2000; Heinrich et al. 2016), practitioners, and patients (Henshaw et al. 2015).

Finally, throughout this study, we have attributed any differences between the younger and older groups to age. However, the older group also had a quantitatively different audiometric profile than the younger group. Given the almost complete absence of overlap in PTA between the two age groups, it is impossible to tease apart the contribution of age versus PTA using statistical tools. On a purely descriptive level, this limitation should not matter too much because age and hearing level are highly confounded in the general population. On a theoretical level, however, if age-related patterns were entirely reducible to a difference in hearing thresholds rather than age, it would mean that poor hearing, rather than age-related cognitive decline, is responsible for an amodal use of processing resources under divided attention. In turn, this would mean that the PTA CL cost observed with the two-back image task in older participants could be lowered, if not eliminated, through aided amplification. A rigorous test of this possibility would involve running the present experiment with younger adults with mild to moderate hearing loss or older adults with good hearing abilities.

## ACKNOWLEDGMENTS

We thank David Maidment for his help with previous versions of the PTA task, and Josh Spowage and Upasana Nathaniel for their help with data collection.

## Supplementary Material



## Footnotes

* Although 3 participants in the older group had high PTA CL cost values, these were not deemed outliers because their raw PTA scores in the NoCL and CL conditions were not outside the conventional 1.5 interquartile range limits.
