# Supplementary material for: Effects of Cognitive Load on Pure-Tone Audiometry Thresholds in Younger and Older Adults
Source: Ear Hear. 2019 Nov 5;41(4):907–17. doi: 10.1097/AUD.0000000000000812 (PMC7676481; doi:10.1097/AUD.0000000000000812)
Supplement: Supplementary file 1 [file aud-41-907-s001.pdf]

Age Group	CL Type	Age	Sex	Baseline PTA					PTA NoCL					PTA CL							SiN					LNS
				.5 KHz	1 KHz	2 KHz	4 KHz	**Avg**	.5 KHz	1 KHz	2 KHz	4 KHz	**Avg**	.5 KHz	1 KHz	2 KHz	4 KHz	**Avg**	**CL Cost**	**CL d'**	2Bab	2SMN	12Bab	12SMM	**Avg SiN**	
Young	Image	22	F	8	0	8	-2	3.5	-2	2	7	3	2.5	-5	7	7	1	2.5	0.0	2.20	-3.61	-10.00	-3.13	-5.75	-5.6	12
		21	M	-4	-1	3	-7	-2.3	0	0	0	-9	-2.3	5	-1	7	-9	0.5	2.8	0.70	-3.06	-10.00	-2.50	-5.63	-5.3	14
		21	F	1	1	-5	-2	-1.3	0	-2	-2	-7	-2.8	5	0	-2	4	1.8	4.5	1.80	-5.25	-7.27	-5.75	-5.25	-5.9	14
		29	M	0	0	-2	-7	-2.3	-3	1	-4	-2	-2.0	-5	-7	-5	0	-4.3	-2.3	1.48	-6.25	-9.72	-1.88	-6.25	-6.0	16
		20	F	0	1	13	8	5.5	-2	0	10	10	4.5	-2	1	11	8	4.5	0.0	1.27	0.00	-10.00	-1.25	-5.00	-4.1	9
		20	M	2	0	2	1	1.3	-2	3	1	3	1.3	0	1	3	3	1.8	0.5	1.52	-2.50	-7.27	-3.13	-5.25	-4.5	17
		19	F	7	3	5	0	3.8	1	-1	-1	-2	-0.8	1	0	2	-5	-0.5	0.3	1.21	-0.63	-4.58	-0.83	-5.63	-2.9	13
		22	F	5	3	1	8	4.3	5	-1	1	5	2.5	3	-2	0	4	1.3	-1.3	1.02	0.00	-7.50	-2.50	-3.25	-3.3	10
		20	F	0	-1	-5	-9	-3.8	-2	-4	-7	-10	-5.8	-2	-2	-7	-10	-5.3	0.5	1.27	-0.28	-10.00	-2.25	-3.75	-4.1	10
		27	F	-4	4	8	-5	0.8	-4	5	8	-4	1.3	-1	5	8	-2	2.5	1.3	1.99	-1.39	-7.08	2.25	-5.45	-2.9	11
		21	F	-5	0	-1	3	-0.8	2	-5	-2	4	-0.3	4	-4	1	3	1.0	1.3	1.12	-4.67	-6.67	-3.06	-5.83	-5.1	10
		23	M	-4	-2	-2	10	0.5	-6	-7	-2	15	0.0	-4	-5	-4	11	-0.5	-0.5	2.15	-5.00	-7.92	-1.25	-6.25	-5.1	14
		26	F	-2	-4	-7	-3	-4.0	0	-7	-7	-5	-4.8	0	-5	-6	-4	-3.8	1.0	1.43	-4.72	-5.63	-2.75	-5.58	-4.7	10
		21	M	-4	-9	-1	-3	-4.3	-8	-10	-3	-9	-7.5	-7	-10	-4	-4	-6.3	1.3	0.96	-3.64	-7.14	-1.25	-6.94	-4.7	14
		21	F	1	-9	0	-2	-2.5	8	-9	-1	5	0.8	7	-10	2	3	0.5	-0.3	1.76	-1.94	-5.63	-3.75	-4.75	-4.0	15
		18	F	8	9	11	5	8.3	5	8	8	7	7.0	1	7	8	6	5.5	-1.5	0.75	-1.88	-4.17	-2.50	-6.75	-3.8	14
		19	F	0	-5	-1	5	-0.3	1	-7	1	1	-1.0	0	-1	0	-2	-0.8	0.3	1.85	-2.27	-7.25	-4.04	-7.50	-5.3	13
		18	M	-1	3	0	-3	-0.3	1	1	3	-3	0.5	3	1	3	-3	1.0	0.5	1.35	-1.82	-9.09	-1.25	-4.75	-4.2	10
		21	M	4	2	28	19	13.3	3	2	31	21	14.3	2	-3	25	18	10.5	-3.8	1.22	-2.75	-7.50	-0.71	-7.50	-4.6	11
		23	F	1	-8	-6	-7	-5.0	-1	-8	-3	3	-2.3	1	-5	-3	1	-1.5	0.8	1.49	-3.75	-7.75	-2.50	-5.91	-5.0	13
		20	F	21	16	-2	20	13.8	23	12	3	29	16.8	19	13	3	25	15.0	-1.8	1.80	-2.32	-6.82	-1.25	-3.75	-3.5	12
		19	F	10	-5	-9	-3	-1.8	12	-5	-9	-7	-2.3	13	-5	-7	-2	-0.3	2.0	1.32	-3.25	-5.00	-3.57	-2.50	-3.6	10
			X	*2.0*	*-0.1*	*1.7*	*1.2*	*1.2*	*1.4*	*-1.5*	*1.5*	*2.2*	*0.9*	*1.7*	*-1.1*	*1.9*	*2.1*	*1.1*	*0.3*	*1.44*	*-2.77*	*-7.45*	*-2.22*	*-5.42*	*-4.5*	*12.4*
			SD	*6.1*	*5.7*	*8.2*	*7.9*	*5.2*	*6.6*	*5.6*	*8.3*	*9.9*	*5.7*	*5.9*	*5.6*	*7.3*	*8.1*	*4.8*	*1.7*	*0.42*	*1.75*	*1.79*	*1.57*	*1.29*	*0.9*	*2.2*

	Rhyme	20	F	8	8	13	6	**8.8**	0	1	8	1	**2.5**	9	4	13	3	**7.3**	**4.8**	**0.53**	2.50	-5.63	-3.27	-2.50	**-2.2**	11
		19	F	-5	1	-4	0	**-2.0**	-7	-4	-5	-1	**-4.3**	-5	-1	-3	0	**-2.3**	**2.0**	**0.91**	-0.28	-6.25	-1.25	-3.75	**-2.9**	10
		19	M	-2	0	1	-10	**-2.8**	-4	-4	-1	-9	**-4.5**	-1	-1	5	-5	**-0.5**	**4.0**	**1.80**	-0.83	-6.25	-1.25	-2.08	**-2.6**	13
		26	M	-1	1	13	7	**5.0**	-2	-3	17	9	**5.3**	1	3	20	9	**8.3**	**3.0**	**0.05**	-0.63	-3.64	-2.86	-1.36	**-2.1**	11
		18	F	6	5	5	3	**4.8**	7	5	5	3	**5.0**	14	5	7	5	**7.8**	**2.8**	**1.17**	-0.71	-4.75	-3.06	-6.25	**-3.7**	14
		26	F	3	1	3	3	**2.5**	1	-3	0	8	**1.5**	1	-2	1	6	**1.5**	**0.0**	**1.15**	-0.42	-2.27	-0.83	-1.94	**-1.4**	14
		21	F	3	1	-3	1	**0.5**	-1	-3	-1	1	**-1.0**	-1	-1	-3	-2	**-1.8**	**-0.8**	**1.34**	-2.50	-6.25	-3.57	-5.63	**-4.5**	14
		31	M	-3	-1	3	5	**1.0**	-2	0	4	3	**1.3**	3	3	7	9	**5.5**	**4.3**	**1.25**	-2.88	-6.88	-3.64	-5.28	**-4.7**	12
		24	F	-2	-2	-2	0	**-1.5**	0	-3	3	10	**2.5**	5	-1	1	8	**3.3**	**0.8**	**1.32**	-2.50	-8.25	-3.75	-4.72	**-4.8**	12
		19	F	18	21	8	1	**12.0**	19	23	5	0	**11.8**	22	24	6	0	**13.0**	**1.3**	**1.11**	-2.75	-7.08	-4.25	-8.61	**-5.7**	11
		21	F	-3	1	5	7	**2.5**	-7	-5	2	6	**-1.0**	-5	-2	4	10	**1.8**	**2.8**	**1.45**	-3.13	-9.72	-4.75	-9.09	**-6.7**	18
		22	F	-6	-10	-2	-7	**-6.3**	-7	-9	-7	-9	**-8.0**	-4	-10	-7	-8	**-7.3**	**0.8**	**1.86**	-1.25	-6.82	-5.63	-4.58	**-4.6**	19
		19	M	6	5	-4	-4	**0.8**	5	1	-2	-7	**-0.8**	8	2	1	-3	**2.0**	**2.8**	**1.06**	-2.50	-4.25	-0.63	-3.25	**-2.7**	15
		21	M	-2	-6	-7	3	**-3.0**	0	-9	-4	0	**-3.3**	1	-7	1	0	**-1.3**	**2.0**	**1.68**	-2.25	-8.64	-4.17	-4.17	**-4.8**	12
		20	F	-5	-5	-4	3	**-2.8**	-7	-7	-7	0	**-5.3**	-6	-5	-6	3	**-3.5**	**1.8**	**1.22**	-5.45	-8.13	-2.50	-3.06	**-4.8**	13
		19	M	-9	-9	-9	-7	**-8.5**	-10	-9	-9	-7	**-8.8**	-7	-9	-10	-6	**-8.0**	**0.8**	**1.17**	-4.55	-7.50	-5.00	-5.75	**-5.7**	16
		23	M	-8	0	-1	2	**-1.8**	-9	-1	1	0	**-2.3**	-9	6	3	1	**0.3**	**2.5**	**1.21**	-1.25	-7.14	-5.63	-5.63	**-4.9**	13
		19	F	25	11	-3	-9	**6.0**	25	13	-2	-9	**6.8**	26	13	-3	-5	**7.8**	**1.0**	**1.38**	-1.25	-5.00	-4.75	-6.82	**-4.5**	12
		18	F	5	-8	-4	-10	**-4.3**	2	-10	-7	-10	**-6.3**	-2	-9	-7	-9	**-6.8**	**-0.5**	**0.99**	-3.27	-4.72	-1.39	-6.25	**-3.9**	11
		18	M	1	-9	-10	-10	**-7.0**	-3	-9	-10	-10	**-8.0**	1	-9	-9	-10	**-6.8**	**1.3**	**1.46**	-1.67	-5.00	-1.39	-5.25	**-3.3**	14
		22	F	-5	-7	8	-7	**-2.8**	-2	-9	3	-10	**-4.5**	-2	-7	5	-7	**-2.8**	**1.8**	**1.83**	-1.25	-8.75	-4.25	-3.13	**-4.3**	12
		20	F	0	-7	6	2	**0.3**	0	-5	8	6	**2.3**	3	-3	10	-4	**1.5**	**-0.8**	**1.32**	-5.67	-2.25	-4.09	-4.17	**-4.0**	20
			X	*1.1*	*-0.4*	*0.5*	*-1.0*	***0.1***	*-0.1*	*-2.3*	*0.0*	*-1.1*	***-0.9***	*2.4*	*-0.3*	*1.6*	*-0.2*	***0.9***	***1.7***	***1.24***	*-2.02*	*-6.14*	*-3.27*	*-4.69*	***-4.0***	*13.5*
			SD	*8.1*	*7.5*	*6.5*	*5.9*	***5.1***	*8.4*	*7.8*	*6.5*	*6.7*	***5.3***	*8.9*	*8.0*	*7.4*	*6.2*	***5.7***	***1.5***	***0.41***	*1.82*	*2.02*	*1.56*	*2.03*	***1.3***	*2.7*
Older	Image	72	M	1	4	14	23	**10.5**	0	3	15	21	**9.8**	3	5	17	26	**12.8**	**3.0**	**1.99**	-3.18	-8.06	-3.65	-4.25	**-4.8**	14
		66	F	1	9	21	14	**11.3**	0	8	25	13	**11.5**	1	11	21	17	**12.5**	**1.0**	**1.40**	-4.09	-6.25	-2.25	-4.75	**-4.3**	17
		78	F	23	15	16	36	**22.5**	24	14	11	31	**20.0**	25	19	13	33	**22.5**	**2.5**	**1.27**	2.50	-4.42	-0.96	-3.18	**-1.5**	14
		66	F	3	6	11	7	**6.8**	5	9	5	1	**5.0**	23	17	22	30	**23.0**	**18.0**	**-0.51**	-1.36	-5.45	-2.50	-3.57	**-3.2**	14
		68	F	33	23	16	18	**22.5**	29	27	18	21	**23.8**	34	26	18	25	**25.8**	**2.0**	**1.68**	-2.14	-7.88	-3.06	-6.25	**-4.8**	10
		66	M	8	11	15	30	**16.0**	8	11	13	25	**14.3**	9	11	11	27	**14.5**	**0.3**	**1.72**	-2.14	-6.25	-1.36	-6.25	**-4.0**	15

	62	F	10	17	21	14	**15.5**	8	17	23	11	**14.8**	15	17	23	15	**17.5**	**2.8**	**1.15**	-3.06	-9.04	-3.06	-5.42	**-5.1**	10
	61	F	5	-1	5	7	**4.0**	5	-1	10	9	**5.8**	3	-7	9	13	**4.5**	**-1.3**	**1.61**	-5.63	-7.86	-3.64	-5.96	**-5.8**	13
	61	M	11	8	15	33	**16.8**	11	5	11	35	**15.5**	11	9	13	35	**17.0**	**1.5**	**1.45**	-4.81	-6.25	-3.06	-4.38	**-4.6**	16
	61	F	8	15	3	19	**11.3**	3	13	9	22	**11.8**	9	15	6	25	**13.8**	**2.0**	**1.62**	-3.25	-7.73	-2.50	-4.72	**-4.6**	14
	63	F	1	1	-4	23	**5.3**	1	-2	-9	23	**3.3**	5	0	-6	29	**7.0**	**3.8**	**1.39**	-1.35	-7.25	-2.32	-4.09	**-3.8**	11
	70	M	-9	-7	1	-5	**-5.0**	-7	-7	3	-2	**-3.3**	-5	-5	5	-7	**-3.0**	**0.3**	**1.54**	-0.63	-5.63	-0.91	-2.88	**-2.5**	13
	84	M	-1	-3	18	48	**15.5**	-1	-4	15	46	**14.0**	1	-1	17	54	**17.8**	**3.8**	**0.24**	1.67	-1.94	1.39	-0.42	**0.2**	8
	64	M	6	-8	21	-1	**4.5**	7	-5	15	16	**8.3**	8	-3	20	4	**7.3**	**-1.0**	**1.07**	-4.17	-8.57	-4.55	-3.57	**-5.2**	16
	63	M	-1	-1	21	16	**8.8**	-1	-3	17	18	**7.8**	-1	4	23	25	**12.8**	**5.0**	**1.81**	-5.71	-10.63	-3.64	-6.25	**-6.6**	10
	74	F	15	14	29	17	**18.8**	19	17	23	14	**18.3**	21	21	25	21	**22.0**	**3.8**	**1.72**	-0.71	-9.42	-2.25	-5.45	**-4.5**	14
	75	F	9	-2	20	33	**15.0**	11	-7	15	26	**11.3**	9	-4	11	30	**11.5**	**0.3**	**1.30**	-3.75	-7.50	-1.82	-5.00	**-4.5**	11
	69	F	15	14	29	17	**18.8**	19	17	23	14	**18.3**	21	21	25	21	**22.0**	**3.8**	**1.28**	-3.06	-7.75	-2.75	-6.25	**-5.0**	13
	66	F	9	-2	20	33	**15.0**	11	-7	15	26	**11.3**	9	-4	11	30	**11.5**	**0.3**	**1.91**	-2.50	-7.27	-3.75	-4.72	**-4.6**	12
	67	F	6	-7	-1	3	**0.3**	8	-3	0	-3	**0.5**	10	-4	1	3	**2.5**	**2.0**	**1.53**	-0.83	-6.88	-1.39	-3.13	**-3.1**	14
	65	F	23	23	23	38	**26.8**	23	19	23	42	**26.8**	21	21	25	41	**27.0**	**0.3**	**1.13**	-1.82	-7.14	-3.13	-5.00	**-4.3**	15
	74	F	3	-2	-5	6	**0.5**	1	-3	-7	9	**0.0**	4	-3	-3	11	**2.3**	**2.3**	**0.01**	-3.04	-6.25	-3.06	-4.72	**-4.3**	12
		X	*8.1*	*5.8*	*14.0*	*19.5*	***11.9***	*8.4*	*5.4*	*12.4*	*19.0*	***11.3***	*10.7*	*7.5*	*14.0*	*23.1*	***13.8***	***2.5***	***1.29***	*-2.41*	*-7.06*	*-2.46*	*-4.56*	***-4.1***	*13.0*
		SD	*9.4*	*9.7*	*10.0*	*13.8*	***8.1***	*9.4*	*10.3*	*9.4*	*12.8*	***7.6***	*9.8*	*10.7*	*9.2*	*13.4*	***8.1***	***3.8***	***0.62***	*2.06*	*1.81*	*1.28*	*1.41*	***1.4***	*2.3*
Rhyme	75	F	8	1	14	35	**14.5**	5	1	15	27	**12.0**	8	4	21	36	**17.3**	**5.3**	**1.11**	-0.63	-6.25	-2.50	-1.82	**-2.8**	15
	82	M	23	18	31	49	**30.3**	23	18	31	55	**31.8**	26	21	31	57	**33.8**	**2.0**	**1.61**	-0.83	-2.86	-1.25	-0.83	**-1.4**	12
	79	M	26	30	28	58	**35.5**	30	33	29	55	**36.8**	31	35	33	61	**40.0**	**3.3**	**0.14**	1.94	0.00	0.83	1.43	**1.1**	10
	69	M	34	27	44	68	**43.3**	31	23	43	66	**40.8**	48	35	48	75	**51.5**	**10.8**	**1.46**	-1.25	-5.25	-1.88	-1.25	**-2.4**	15
	67	M	8	9	21	21	**14.8**	10	9	23	21	**15.8**	11	7	25	29	**18.0**	**2.3**	**1.62**	-1.67	-6.25	-2.88	-5.63	**-4.1**	17
	71	M	9	16	17	20	**15.5**	8	13	20	21	**15.5**	8	15	19	22	**16.0**	**0.5**	**1.31**	-3.75	-8.75	-4.29	-5.91	**-5.7**	14
	72	M	0	2	15	49	**16.5**	-4	3	13	43	**13.8**	21	11	15	53	**25.0**	**11.3**	**0.69**	-6.25	-4.75	-2.14	-5.00	**-4.5**	14
	66	F	23	21	13	26	**20.8**	23	21	15	30	**22.3**	25	24	13	29	**22.8**	**0.5**	**1.23**	-3.06	-6.75	-5.00	-5.75	**-5.1**	9
	62	F	6	13	8	15	**10.5**	5	6	8	16	**8.8**	4	9	5	13	**7.8**	**-1.0**	**1.27**	-3.25	-6.25	-4.17	-5.63	**-4.8**	11
	66	F	19	11	5	24	**14.8**	18	9	3	18	**12.0**	18	12	1	21	**13.0**	**1.0**	**1.61**	-1.35	-5.91	-3.61	-5.00	**-4.0**	11
	62	M	8	-8	1	7	**2.0**	7	-4	5	11	**4.8**	8	2	5	16	**7.8**	**3.0**	**1.58**	-4.04	-7.86	-5.63	-6.39	**-6.0**	13
	63	M	-1	-4	7	9	**2.8**	-1	-4	1	3	**-0.3**	-3	3	2	0	**0.5**	**0.8**	**1.00**	-4.38	-8.75	-3.57	-6.88	**-5.9**	19

61	M	0	-4	18	22	**9.0**	1	-5	19	23	**9.5**	3	-4	17	23	**9.8**	**0.3**	**1.49**	-2.75	-7.86	-0.83	-4.58	**-4.0**	11
70	F	5	5	21	10	**10.3**	8	5	23	11	**11.8**	9	1	19	17	**11.5**	**-0.3**	**0.00**	-3.06	-6.75	-1.82	-6.75	**-4.6**	13
63	M	1	-6	3	25	**5.8**	-2	-7	6	23	**5.0**	4	-1	7	23	**8.3**	**3.3**	**1.17**	-1.25	-6.00	-3.06	-5.63	**-4.0**	14
63	F	0	-4	1	20	**4.3**	-5	5	5	24	**7.3**	-3	3	1	26	**6.8**	**-0.5**	**1.58**	-2.92	-6.39	-4.17	-6.36	**-5.0**	17
71	F	28	30	26	23	**26.8**	27	28	24	26	**26.3**	30	25	23	25	**25.8**	**-0.5**	**0.88**	-3.33	-5.00	-4.75	-5.00	**-4.5**	14
65	F	16	3	17	25	**15.3**	23	3	9	21	**14.0**	17	5	17	23	**15.5**	**1.5**	**1.07**	-5.63	-7.08	-4.29	-6.25	**-5.8**	13
60	F	3	1	1	10	**3.8**	1	-1	1	8	**2.3**	6	5	1	6	**4.5**	**2.3**	**1.30**	-4.17	-8.06	-4.09	-5.63	**-5.5**	12
62	M	17	-5	11	39	**15.5**	16	-3	10	36	**14.8**	15	1	15	43	**18.5**	**3.8**	**0.96**	-1.36	-3.33	-0.63	-2.12	**-1.9**	16
71	F	35	36	36	57	**41.0**	26	28	41	52	**36.8**	30	31	38	56	**38.8**	**2.0**	**0.69**	5.00	3.13	2.92	0.28	**2.8**	13
74	M	21	61	61	71	**53.5**	19	53	63	68	**50.8**	23	57	63	60	**50.8**	**0.0**	**2.33**	4.55	5.25	6.75	3.75	**5.1**	7
	X	*13.1*	*11.5*	*18.1*	*31.0*	***18.5***	*12.2*	*10.6*	*18.5*	*29.9*	***17.8***	*15.4*	*13.7*	*19.0*	*32.5*	***20.1***	***2.3***	***1.19***	*-1.97*	*-5.08*	*-2.28*	*-3.95*	***-3.3***	*13.2*
	SD	*11.5*	*17.2*	*15.1*	*19.4*	***14.2***	*11.7*	*15.2*	*15.6*	*18.7*	***13.7***	*12.8*	*15.3*	*16.0*	*19.9*	***14.6***	***3.2***	***0.51***	*2.83*	*3.63*	*2.86*	*2.98*	***2.9***	*2.8*
